# Tumor de corpo carotídeo (paraganglioma): relato de dois casos submetidos a tratamento cirúrgico

**DOI:** 10.1590/1677-5449.007315

**Published:** 2016

**Authors:** Nelson Mesquita, Rogério Santos Silva, José Henrique Agner Ribeiro, Lislaine Cruz Batista, Emanuelle Melania Stedille Bringhentti, Bruno Benjamin Brunini de Souza, Lisiane Cristine da Mota Cabral

**Affiliations:** 1 Faculdade Evangélica do Paraná – FEPAR, Curitiba, PR, Brasil.

**Keywords:** tumor de corpo carotídeo, paraganglioma, tumor de glomo carotídeo

## Abstract

O tumor de corpo carotídeo é uma neoplasia rara, geralmente benigna, que acomete, sobretudo, indivíduos entre a quarta e a quinta décadas de vida. Manifesta-se pela presença de massa cervical consistente localizada abaixo do ângulo da mandíbula, pulsátil e comumente indolor. Pode evoluir para dor local, disfagia, soluços, rouquidão e síndrome do corpo carotídeo hipersensível. Este artigo relata os casos de duas pacientes diagnosticadas com essa neoplasia e submetidas ao tratamento cirúrgico. A primeira foi submetida a uma ressecção em bloco do tumor, enquanto a segunda, com estadiamento mais precoce, foi tratada com uma ressecção subadventicial da lesão.

## INTRODUÇÃO

O corpo carotídeo é uma estrutura de formato elíptico de 3 a 4 mm de tamanho localizada na bifurcação da carótida comum ao nível da sua camada adventicial^1^^-^ 4. Tem como função a quimiorrecepção e a barorrecepção. Por essa razão, os tumores provenientes desse local são chamados de quimiodectomas, isto é, tumores quimiorreceptores^2^^,^ 5 ^-^ 7.

Os tumores de corpo carotídeo são neoplasias derivadas de células paragangliônicas e, apesar de serem bem delimitados, são tumores não capsulados e altamente vascularizados por ramos da artéria carótida externa e seus “vasa vasorum”^2^^-^ 4 ^,^ 8 ^-^ 12.

Apesar de raros, são os paragangliomas mais comuns originados na região da cabeça e pescoço (60-70%)^5^^,^ 13. Essa neoplasia é, em grande parte das vezes, hipervascularizada, de origem familiar e crescimento lento, e não há predisposição por sexo. Acomete, sobretudo, pacientes na quarta e quinta décadas de vida, sendo, na grande maioria das vezes, de caráter benigno; porém, grande parte dos autores relatam malignidade em 5 a 6% dos casos^1^^,^ 6 ^,^ 14 ^,^ 15. Os sintomas são variados. Pode ser assintomático ou manifestar-se como uma tumoração de crescimento lento, indolor e pulsátil na região lateral do pescoço, próximo ao ângulo da mandíbula, com eventual queixa de rouquidão, dificuldade de deglutição e sintomas da síndrome do seio carotídeo^5^^,^ 6 ^,^ 16.

## DESCRIÇÃO DOS CASOS

**Figura 1:**
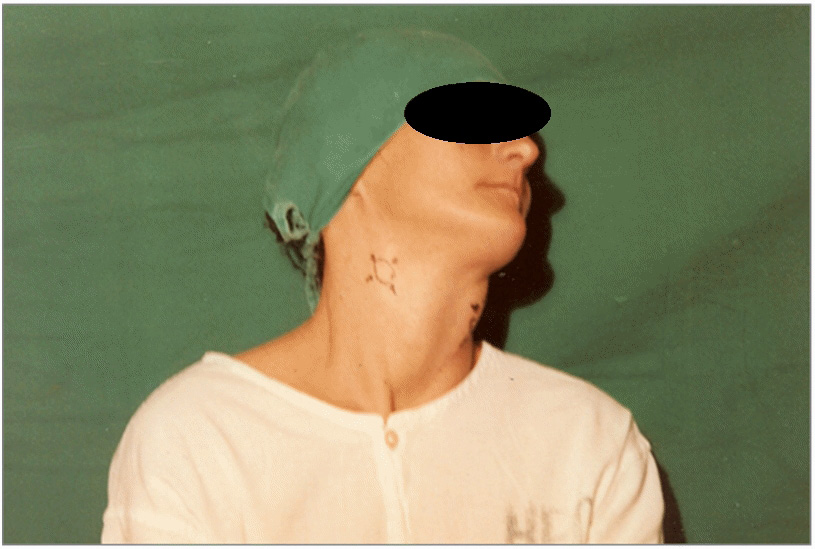
Presença de lesões tumorais, bilateralmente, em região topográfica correspondente aos vasos carotídeos.

A primeira paciente, 28 anos, sexo feminino, branca, natural de Loanda (PR), foi atendida no ambulatório do Hospital Universitário Evangélico de Curitiba (HUEC) com história de tumor em região cervical bilateralmente, com evolução de 5 anos, sendo submetida 4 meses antes a biópsia cirúrgica na região cervical esquerda e encaminhada com diagnóstico de aneurisma de carótida. Negava antecedentes de disfagia, disfonia, ataques isquêmicos e emagrecimento. Ao exame físico, foi observada uma cicatriz de aproximadamente 3 cm no bordo anterior do músculo esternocleidomastoideo, à esquerda. Através da palpação, ficaram evidentes duas massas ovoides, uma medindo 3 × 4 cm, à esquerda, e outra medindo 2 × 2 cm, à direita (Figura 1). Ambas se localizavam nos triângulos carotídeos direito e esquerdo e apresentavam uma consistência elástica, indolor, pulsátil, com mobilidade lateral, porém fixas no sentido longitudinal, sem frêmitos e sopros. O exame da cavidade oral não evidenciou lesões e adenopatias em outras regiões. O exame neurológico mostrou integridade dos pares cranianos.

**Figura 2:**
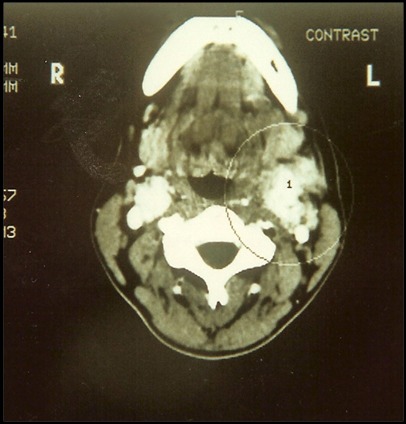
Imagem tomográfica que deixa evidente a presença de lesão tumoral ao nível da bifurcação carotídea, bilateralmente.

**Figura 3:**
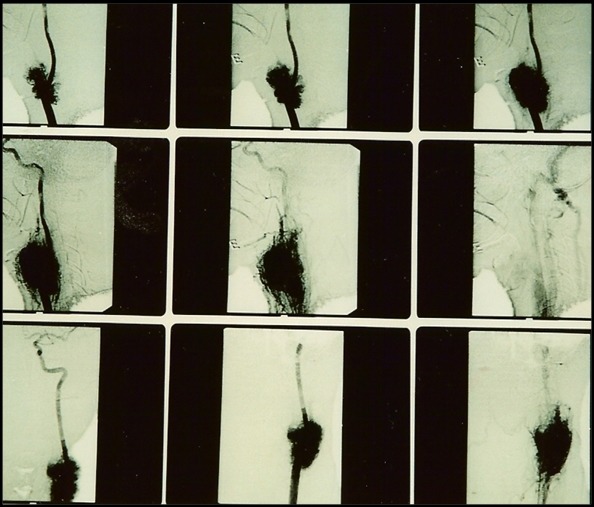
Exame arteriográfico através do qual se pode observar a rica vascularização tumoral, além da ausência de enchimento da carótida externa pelo contraste.

A paciente foi hospitalizada e submetida a exames de eco-Doppler colorido e tomografia computadorizada (Figura 2), que sugeriram diagnóstico de tumor de corpo carotídeo, posteriormente confirmado pela arteriografia (Figura 3). À esquerda, observou-se oclusão da carótida externa na origem. O exame tomográfico também descartou sinais de invasão da base do crânio.

**Figura 4:**
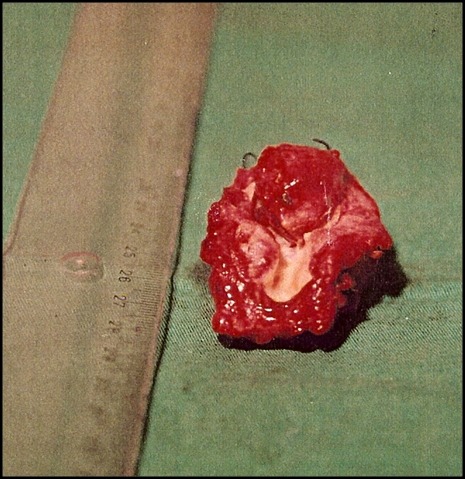
Aspecto da peça cirúrgica, produto da ressecção em bloco do tumor juntamente com a bifurcação carotídea.

**Figura 5:**
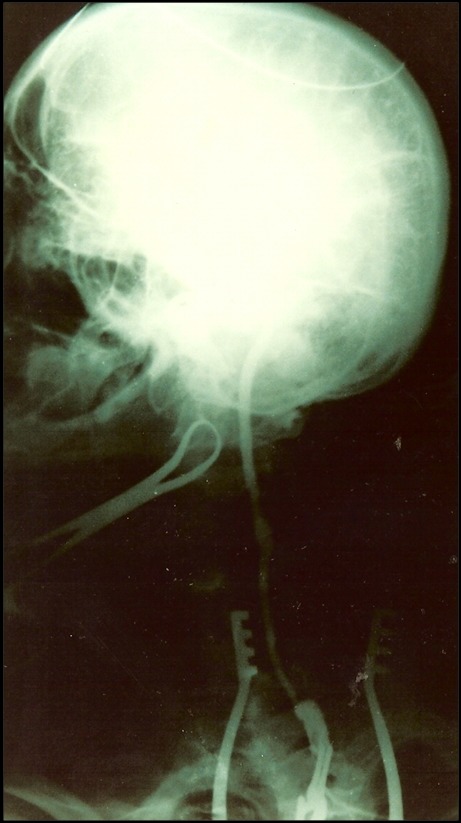
Arteriografia transoperatória de controle que mostra a perviedade da veia safena usada na reconstrução.

**Figura 6:**
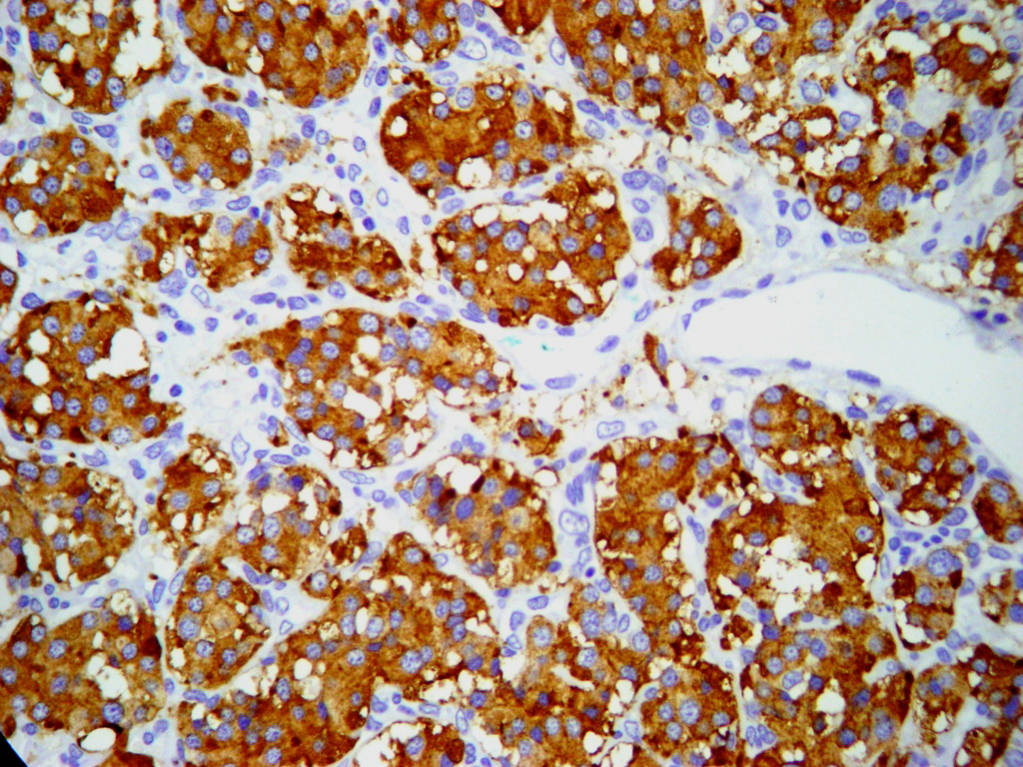
Exame histopatológico que, através da técnica de imuno-histoquímica, deixa clara a presença das células cromafins.

**Figura 7:**
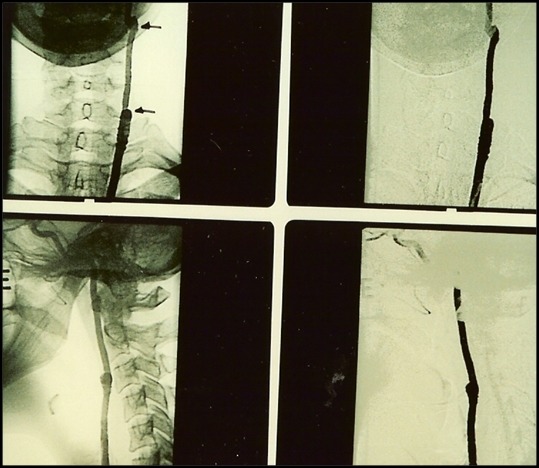
Arteriografia de controle após 60 dias, que não mostrou resquícios tumorais.

A paciente foi submetida a cirurgia sob anestesia geral. Realizou-se uma incisão no bordo anterior do músculo esternocleidomastoideo esquerdo, encontrando-se uma tumoração sólida aderida à bifurcação carotídea, sem comprometer os nervos vago e hipoglosso. Observou-se ligadura da carótida externa na origem. Devido à dificuldade na dissecção, foi realizada a ressecção em bloco do tumor e da bifurcação carotídea (Figura 4). Utilizou-se *shunt* temporário de Pruitt-Inahara para proteção cerebral, interposição de safena interna com anastomose proximal lateroterminal e distal terminoterminal (Figura 5). Foram retirados dois gânglios para análise histopatológica. A paciente evoluiu no pós-operatório sem déficit neurológico, recebendo alta no sexto dia. O exame histopatológico confirmou diagnóstico de tumor de corpo carotídeo com gânglios livres (Figura 6). Após 60 dias de pós-operatório, um novo estudo arteriográfico não evidenciou resquícios do tumor (Figura 7). A cirurgia do lado contralateral não foi realizada por recusa da paciente.

A segunda paciente, do sexo feminino, 65 anos, natural de Curitiba (PR), queixava-se da presença de tumoração na região cervical esquerda. Relatava histórico de hipertensão arterial sistêmica e hipercolesterolemia, ambas sob controle. A paciente também relatou possuir meningioma aderido ao seio sagital superior, que não apresentava atividade de crescimento desde 2009. Ao exame físico, verificou-se, na palpação, a presença de massa ovoide pulsátil e indolor em trígono carotídeo esquerdo. Havia consistência elástica sem aderência aos planos profundos e com mobilidade lateral, porém imobilidade em sentido longitudinal. Sem frêmito e sopros. Assim como no caso anterior, os exames da cavidade oral e neurológico não mostraram alterações. A paciente foi submetida a exames de imagem no período pré-operatório. O eco-Doppler colorido evidenciou massa tumoral extremamente vascularizada com dimensões de 3,9 × 3,2 cm ao nível na bifurcação carotídea com afastamento das carótidas internas e externa (“sinal da lira”).

**Figura 8:**
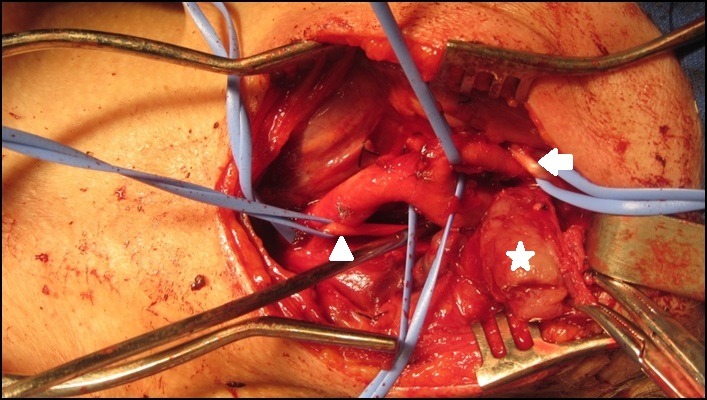
Ressecção de tumor glômico através da técnica subadventicial. Nesta imagem, podem-se observar as seguintes estruturas sendo isoladas dos vasos carotídeos: nervo vago (ponta de flecha); nervo hipoglosso (flecha); e tumor (estrela).

O tratamento cirúrgico foi instituído. Sob anestesia geral, fez-se uma incisão cervical semelhante à descrita no caso anterior, à esquerda. Foi encontrada uma massa sólida aderida à bifurcação carotídea. As estruturas nobres adjacentes foram isoladas facilmente e a dissecção do tumor foi realizada pela técnica subadventicial sem intercorrências (Figuras 8 e 9). Retirou-se material para análise histopatológica. Foi colocado um dreno suctor durante as primeiras 24 horas de pós-operatório. A paciente evoluiu no pós-operatório sem complicações e recebeu alta no segundo pós-operatório. O exame histopatológico confirmou diagnóstico de paraganglioma carotídeo.

**Figura 9:**
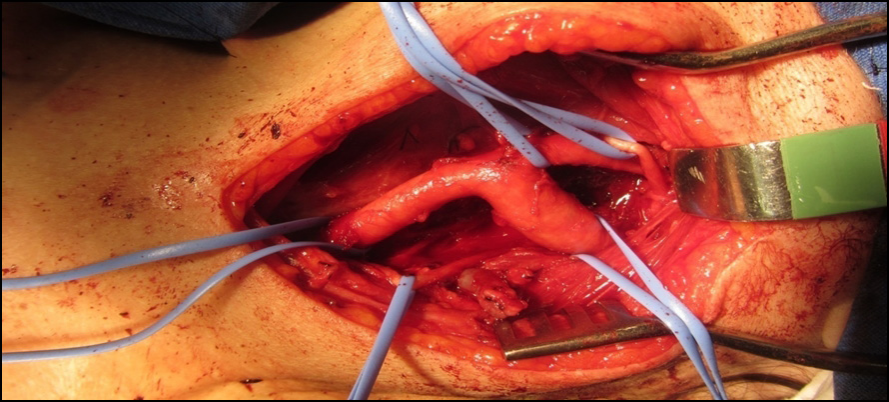
Aspecto do sítio cirúrgico após a ressecção.

## DISCUSSÃO

Segundo Shamblin et al.^3^, o tumor de corpo carotídeo é classificado em três grupos de acordo com a extensão circunferencial e o grau de aderência: Grupo I - tumor pequeno sem aderência aos vasos que pode ser ressecado sem causar danos às estruturas vizinhas; Grupo II - tumor intermediário com pequena aderência aos vasos cuja dissecação é mais difícil, sendo necessária, às vezes, a revascularização; Grupo III - tumor grande com infiltração nos vasos cuja dissecação se torna quase impraticável, sendo necessária a ressecção em bloco com bifurcação carotídea e realização de revascularização com veia safena ou prótese^6^^,^ 17 ^,^ 18.

Em relação aos exames complementares, o eco-Doppler colorido é a primeira opção, pois fornece informações sugestivas do diagnóstico, que são importantes para triagem e diagnóstico diferencial. A tomografia computadorizada cervical ou, melhor ainda, a ressonância nuclear magnética são os exames de escolha para obter dados de localização, extensão, correlação com estruturas adjacentes e natureza vascular do tumor^6^^,^ 12 ^,^ 13 ^,^ 19. A cintilografia de corpo inteiro e a citometria de fluxo de DNA podem ser usadas na pesquisa de lesões metastáticas ou tumores múltiplos^17^^,^ 20. Já a biópsia não é indicada devido à grande chance de sangramento^5^^,^ 10 ^,^ 12.

A cirurgia é o tratamento de escolha, tendo em vista a possibilidade de malignização, invasão peritumoral e metastação. A técnica mais utilizada é a dissecção subadventicial do tumor (Gordon-Taylor)^17^^,^ 18 ^,^ 21. A ressecção em bloco é utilizada apenas nos casos em que é inviável separar o tumor da artéria, e deve ser seguida de interposição de um enxerto para a carótida interna^17^. Pantanowitz et al., em 1984, ao fazer uma comparação entre o tamanho do tumor e a classificação de Shamblin, percebeu que os tumores de até 6 cm de tamanho correspondem aos grupos 1 e 2 de Shamblin, e os maiores de 6 cm correspondem ao grupo 3. Portanto, concluiu que os tumores que têm indicação de dissecção são os que possuem menos de 6 cm e extensão circunferencial incompleta, com ângulo da bifurcação menor que 90º, e os que devem ser abordados por ressecção são os maiores de 6 cm e extensão circunferencial completa, com ângulo da bifurcação maior que 90º^18^^,^ 22. O tumor é considerado inoperável quando envolve toda a carótida interna extracraniana, o que torna impossível a anastomose distal com enxerto ou prótese^10^.

No intraoperatório, é muito importante, antes da dissecção, a identificação e a exposição ampla de todos os nervos, evitando sua manipulação em excesso^5^^,^ 23. Em certas situações, como a ressecção em bloco ou uma possível lesão da parede arterial, faz-se necessário o uso de *shunt* 17. Barbitúricos e manitol são preconizados quando se necessita clipar a artéria carótida interna^24^.

As taxas de complicações gerais variam entre 32 e 44%, e as taxas de mortalidade variam entre 8 e 20%^25^. A maior causa de morbidade é a lesão de pares cranianos, sendo os mais acometidos o hipoglosso, o vago e o laríngeo superior, que ocasionam paralisias muitas vezes definitivas^4^^,^ 17 ^,^ 18 ^,^ 23. Complicações mais graves, como infarto cerebral e hemorragia de difícil controle, são mais raras^4^.

A embolização pode ser utilizada no caso de tumores grandes, em que ela reduz a perda sanguínea e o tamanho do tumor e melhora os resultados cirúrgicos, porém não se pode descartar o risco de embolia cerebral^17^^,^ 18. A radioterapia pode ser utilizada para tumores inacessíveis, parcialmente ressecados, metastáticos, em casos de recorrência local e nos pacientes em que a cirurgia tem altos índices de morbidade. Porém, é necessário lembrar que efeitos colaterais podem ocorrer, como necrose da mandíbula, cérebro e partes moles^17^^,^ 18. A pesquisa do tumor de corpo carotídeo é indicada para os familiares de primeiro grau, e os pacientes devem ser acompanhados em longo prazo, pois a doença metastática pode levar de 10 a 20 anos para se tornar evidente^6^.
