# Supplementary material for: Tumor de corpo carotídeo (paraganglioma): relato de dois casos submetidos a tratamento cirúrgico
Source: J Vasc Bras. 2016 Apr-Jun;15(2):158–64. [Article in Portuguese] doi: 10.1590/1677-5449.007315 (PMC5829712; doi:10.1590/1677-5449.007315)
Supplement: Translation

# Translation: Carotid body tumor (paraganglioma): report of two cases treated surgically

**Authors:** Nelson Mesquita, Rogério Santos Silva, José Henrique Agner Ribeiro, Lislaine Cruz Batista, Emanuelle Melania Stedille Bringhentti, Bruno Benjamin Brunini de Souza, Lisiane Cristine da Mota Cabral

**Affiliations:** 1 Faculdade Evangélica do Paraná – FEPAR, Curitiba, PR, Brazil.

**Keywords:** carotid body tumor, paraganglioma, carotid glomus tumor

## Abstract

A carotid body tumor is a rare neoplasm, generally benign, that predominantly affects people between their fourth and fifth decades of life. It manifests as a pulsatile and generally painless cervical mass with firm consistency, located below the angle of the jaw. It can progress to the extent that it causes localized pain, dysphagia, hiccups, hoarseness and hypersensitive carotid body syndrome. This article reports the cases of two female patients diagnosed with this tumor who were treated surgically. The first was treated with block resection of the tumor, while the second patient, who had an early stage tumor, was treated with subadventitial resection of the lesion.

## INTRODUCTION

The carotid body is a structure of elliptical shape, 3 to 4 mm in size, that is located at the bifurcation of the common carotid, at the level of its adventitial layer.^1^^-^ 4 Its functions are chemoreception and baroreception and because of this tumors that originate from it are called chemodectomas, i.e., chemoreceptor tumors.^2^^,^ 5 ^-^ 7

Carotid body tumors are neoplasms that arise from paraganglion cells and although they are well-delimited, they are unencapsulated tumors and are highly vascularized by branches of the external carotid artery and their “vasa vasorum”.^2^^-^ 4 ^,^ 8 ^-^ 12

While rare, they are the most common paragangliomas that occur in the head and neck area (60-70%).^5^^,^ 13 In the great majority of cases these neoplasms are hypervascularized, of familial origin and slow growing, and there is no predilection for either sex. They predominantly occur in patients in their fourth and fifth decades of life and the great majority are benign; however, a large proportion of authors report malignancy in 5 to 6% of cases.^1^^,^ 6 ^,^ 14 ^,^ 15 Symptoms are varied. They can be asymptomatic or manifest as a slow-growing tumor that is painless and pulsatile, in the side of the neck, close to the angle of the jaw, and occasionally lead to complaints of hoarseness, deglutition problems and symptoms of carotid sinus syndrome.^5^^,^ 6 ^,^ 16

## DESCRIPTIONS OF CASES

The first patient was a 28-year-old white female from Loanda, PR, Brazil, who was seen at a clinic in the Hospital Universitário Evangélico de Curitiba with a history of bilateral tumors in the cervical region, with onset 5 years previously. She had had a surgical biopsy of the left cervical region 4 months previously and referred with a diagnosis of carotid aneurysm. She stated that she had no history of dysphagia, dysphonia, ischemic attacks or weight loss. During the physical examination, a scar approximately 3 cm long was observed at the anterior margin of the sternocleidomastoid muscle, on the left. Palpation revealed two ovoid masses, one measuring 3 x 4 cm, on the left, and the other measuring 2 x 2 cm, on the right (Figure 1). They were located within the right and left carotid triangles and had an elastic consistency, were painless and pulsatile and were mobile in the lateral direction, but immobile longitudinally. They did not produce thrills or murmurs. Examination of the oral cavity did not find evidence of lesions or adenopathies in other sites. The neurological examination showed that the cranial pairs were undamaged.

**Figure 1:** Presence of bilateral tumors in topographic area corresponding to the carotid vessels.

The patient was admitted to hospital and underwent examinations with color Doppler ultrasonography and computed tomography (Figure 2), which were suggestive of carotid body tumor and this diagnosis was later confirmed by arteriography (Figure 3). On the left, it could be observed that the external carotid was occluded at the origin. The tomographic examination ruled out signs of invasion of the base of the skull.

**Figure 2:** Tomographic image showing evidence of the presence of bilateral tumors at the level of the carotid bifurcations.

**Figure 3:** Arteriographic examination in which the rich tumoral vascularization can be seen, in addition to absence of contrast filling the external carotid.

The patient underwent surgery under general anesthesia. An incision was made at the anterior margin of the left sternocleidomastoid muscle, revealing a solid tumor adhering to the carotid bifurcation, without involving the vagus or hypoglossal nerves. A ligature of the external carotid at the origin was observed. Since dissection was difficult, en bloc resection of the tumor and carotid bifurcation was performed (Figure 4). A temporary Pruitt-Inahara shunt was used for cerebral protection, with interposition of the internal saphenous with proximal side-to-end anastomosis and distal end-to-end anastomosis (Figure 5). Two glands were removed for histopathological analysis. The patient recovered during the postoperative period with no neurological deficits and was discharged on the sixth day. The histopathological analysis confirmed the diagnosis of carotid body tumor and found the glands to be free of involvement (Figure 6). A repeat arteriography conducted 60 days after the procedure did not find evidence of tumor remnants (Figure 7). The patient refused surgery on the contralateral tumor.

**Figure 4:** Surgical specimen, product of resection en bloc of tumor together with the carotid bifurcation.

**Figure 5:** Transoperative control arteriography, showing patency of the saphenous vein used for reconstruction.

**Figure 6:** Histopathological analysis with immunohistochemical technique, clearly showing the presence of chromaffin cells.

**Figure 7:** Control arteriography at 60 days, which does not show tumor remnants.

The second patient was a 65-year-old female from Curitiba, PR, Brazil, who complained of a tumor in the left cervical region. She reported a history of systemic arterial hypertension and hypercholesterolemia, both under control. She also reported having a meningioma adhering to the superior sagittal sinus, which had not exhibited growth since 2009. During physical examination, palpation identified an ovoid, pulsatile, and painless mass in the left carotid trigone. It was of elastic consistency, with no adherence to the deeper planes and was mobile laterally, but immobile in the longitudinal direction. There was no thrill or murmur. In common with the first case, examination of the oral cavity and a neurological examination did not detect abnormalities. The patient underwent imaging exams during the preoperative period. Color Doppler ultrasonography showed an extremely vascularized tumor with dimensions of 3.9 × 3.2 cm at the level of the carotid bifurcation, with separation of the internal and external carotids (the “lyre sign”).

Surgical treatment was chosen. Under general anesthesia, a cervical incision similar to that described in the previous case description was made on the left. A solid mass was found adhering to the carotid bifurcation. Essential adjacent structures were isolated easily and the tumor dissected using the subadventitial technique with no intercurrent events (Figures 8 and 9). Material for histopathological analysis was removed. A Jackson-Pratt drain was used for the first 24 postoperative hours. The patient recovered well during the postoperative period, with no complications, and was discharged on the second postoperative day. Histopathological analysis confirmed the diagnosis of carotid paraganglioma.

**Figure 8:** Resection of the glomus tumor using the subadventitial technique. This image shows the following structures being isolated from the carotid vessels: vagus nerve (arrowhead); hypoglossal nerve (arrow); and tumor (star).

**Figure 9:** Appearance of surgical site after resection.

## DISCUSSION

According to Shamblin et al.^3^ carotid body tumors can be classified into three groups depending on their circumference and degree of adherence: Group I – small tumor with no adherence to vessels that can be resected without damaging neighboring structures; Group II – intermediate tumor with minor adherence to vessels, which is more difficult to dissect and may require revascularization; Group III – large tumor with infiltration of vessels, which is almost impractical to dissect, making resection en bloc with the carotid bifurcation and revascularization with saphenous vein or prosthetic graft necessary.^6^^,^ 17 ^,^ 18

With regard to work-up tests and examinations, color Doppler ultrasonography is the first choice, because it provides information that is suggestive of the diagnosis and important for screening and differential diagnosis. Cervical computed tomography or, better still, magnetic resonance imaging, are the examinations of choice for obtaining data on location, extension, correlation with adjacent structures and vascular nature of the tumor.^6^^,^ 12 ^,^ 13 ^,^ 19 Whole-body scintigraphy and DNA flow cytometry can be used to investigate the possibility of metastatic lesions or multiple tumors.^17^^,^ 20 Biopsy is not indicated because of the considerable chance of bleeding.^5^^,^ 10 ^,^ 12

Surgery is the treatment of choice, bearing in mind the possibility of malignant transformation, peritumoral invasion and metastasis. The most widely-used technique is subadventitial dissection of the tumor (Gordon-Taylor).^17^^,^ 18 ^,^ 21 Resection en bloc is only employed in cases in which it is not feasible to separate the tumor from the artery, and should be followed by interposition of a graft to the internal carotid.^17^ In 1984, Pantanowitz et al. conducted a comparison between size of tumor and Shamblin grades, finding that tumors up to 6 cm in size corresponded to Shamblin grades 1 and 2 and those larger than 6 corresponded to group 3. They therefore concluded that tumors that were smaller than 6 cm and had an incomplete circumferential extension and an angle of bifurcation less than 90º were indicated for dissection and that those that should be treated with resection were larger than 6 cm and with complete circumferential extension and an angle of bifurcation greater than 90º.^18^^,^ 22 Tumors are considered inoperable if they surround the entire extracranial internal carotid, which makes it impossible to perform distal anastomosis with graft or prosthesis.^10^

During the procedure, before dissection, it is very important to identify and fully expose all nerves, avoiding excessive handling.^5^^,^ 23 In certain situations, such as resection en bloc or a possible injury to the artery wall, it will be necessary to construct a shunt.^17^ Barbiturates and mannitol are recommended if it is necessary to clamp the internal carotid artery.^24^

Overall complication rates vary from 32 to 44% and mortality rates vary from 8 to 20%.^25^ The greatest cause of morbidity is injury to the cranial pairs, of which the hypoglossal, vagus and laryngeal superior are the most often affected, which can cause paralysis that is very often definitive.^4^^,^ 17 ^,^ 18 ^,^ 23 More serious complications, such as strokes and difficult to control hemorrhages, are rarer.^4^

Embolization can be employed in cases of large tumors, which will reduce blood loss and the size of the tumor and improve the results of surgery, but the risk of cerebral embolism cannot be ruled out.^17^^,^ 18 Radiotherapy can be used for inaccessible, partially resected and/or metastatic tumors, in cases of local recurrence and in patients in whom surgery has high rates of morbidity. However, it must be remembered that side effects can occur, such as necrosis of the jaw, brain and soft tissues.^17^^,^ 18 Screening for carotid body tumors is indicated for first-degree relatives and patients should receive long-term follow-up, because metastatic disease can take from 10 to 20 years to appear.^6^
